# Titanium Dioxide Nanoparticles-Induced Genotoxic Effects in Mosquito *Culex quinquefaciatus*

**DOI:** 10.3390/toxics12120871

**Published:** 2024-11-29

**Authors:** Aastha Saini, Kanwaljit Kaur Ahluwalia, Amrik Singh Ahluwalia, Neelam Thakur, Puneet Negi, Abeer Hashem, Khalid F. Almutairi, Elsayed Fathi Abd_Allah

**Affiliations:** 1 Department of Zoology, Akal College of Basic Sciences, Eternal University, Baru Sahib, Sirmaur 173101, Himachal Pradesh, India; aasthasaini052002@gmail.com; 2 Department of Botany, Akal College of Basic Sciences, Eternal University, Baru Sahib, Sirmaur 173101, Himachal Pradesh, India; amrik.s511@gmail.com; 3 Department of Physics, Akal College of Basic Sciences, Eternal University, Baru Sahib, Sirmaur 173101, Himachal Pradesh, India; puneetnegiphyrnd@gmail.com; 4 Botany and Microbiology Department, College of Science, King Saud University, P.O. Box. 2460, Riyadh 11451, Saudi Arabia; habeer@ksu.edu.sa; 5 Plant Production Department, College of Food and Agricultural Sciences, King Saud University, P.O. Box 2460, Riyadh 11451, Saudi Arabia; almutairik@ksu.edu.sa (K.F.A.); eabdallah@ksu.edu.sa (E.F.A.)

**Keywords:** titanium dioxide nanoparticles, mitotic index, genotoxic effect, LD_20_, *Culex quinquefaciatus*, chromosomal aberrations

## Abstract

Titanium dioxide (TiO_2_) nanoparticles are being extensively used in a wide range of industrial applications for producing a variety of different consumer products, including medicines and even food items. The consumption of these products is increasing at an alarming rate, and this results in the release of these nanoparticles in the environment, causing a threat to organisms thriving in aquatic as well as terrestrial ecosystems. That is why screening such materials for their genotoxic effects, if any, becomes essential. A toxicity assay was performed to determine the LD_20_ of these nanoparticles for the mosquito *Culex quinquefaciatus* by Probit analysis. Early fourth instar larvae were exposed to the selected dose of 50 µg/mL, which is <LD_20_ value, for 24 h treatment. Chromosomal slides were prepared from lacto-aceto-orcein-stained gonads of adult mosquitoes developed from treated and control larvae. These nanoparticles were reported cytotoxic as a statistically significant decline in mitotic index in treated mosquitoes than controls were observed. The nanoparticles were also found to induce various structural and numerical chromosomal aberrations in the treated lot. In the end, it can be concluded that these nanoparticles do have a genotoxic effect. The present study provides a caution against further use of these nanoparticles. There must be the development of strategies for the safe, sustainable use as well as proper disposal of these nanoparticles so as to protect both biotic and non-biotic components of the environment.

## 1. Introduction

Nanomaterials have been implemented in various industrial and agricultural sectors for decades. Due to their ever-increasing commercial applications and presence in the majority of consumer products, these are being extensively investigated for their safety. Genotoxicity, which is the tendency of physical and chemical agents to alter genetic information, is one of the major concerns with respect to nanomaterials [1].

TiO_2,_ often known as titania, was first mass-produced in the early 20th century as a non-toxic alternative to a white dye for paint. Later, it and its nano-forms use were extended to diverse commercial products, and now it can be found in all kinds of toothpaste, ceramics, printing inks, paper, plastics, rubber, synthetic fibers, condensers, paints and crayons, electronic components along with food and cosmetics [2,3,4,5,6,7,8,9]. In addition to these utilities, more and more photodynamic implementation trials are using TiO_2_ NPs with the main focus on their significance as photosensitizing agents in the treatment of malignant tumors as well as in the photodynamic inactivation of antibiotic-resistant bacteria [10]. These have also been used in photoelectrochemical bio-sensing applications that greatly improve target detection. That is why these are often loaded with various medications and employed as local drug delivery systems for various diseases such as osteoporosis, cancer, and microbial inflammations [11].

Various experimental and epidemiological studies have reported severe effects of titanium dioxide nanoparticles in the liver, brain, kidney and spleen, causing adverse impacts on human health [12,13,14,15]. These are also reported to be neurotoxic, resulting in memory loss and compromised learning capabilities in rodents [16,17,18]. These particles are classified as group 2B carcinogens (suspected carcinogens) by the International Agency for Research on Cancer. In 2021, the European Food Safety Authority (EFSA) declared TiO_2_-NPs unsafe for their use as food additives as their long-time exposure and high dosage could affect the function of various major organs and recommended even their ban for further use. A ban on titanium dioxide (E171) as a food additive was implemented in the European Union in 2022 as its use is simply to enhance the aesthetic value of food only and does not serve any important purpose. On the one hand, these nanoparticles are being used in diverse applications; on the other hand, these are reported to confer toxicity because of their undesired interactions with many biological components and cellular processes [19,20,21]. In the present scenario, the genotoxicity of TiO_2_ NPs to living organisms is a topic of high concern and needs proper assessment. Different researchers have already obtained controversial results as far as the genotoxicity of these NPs is concerned using different endpoints in various in vitro and in vivo test models [22,23,24]. In view of these contrasting results, the present piece of research work is carried out particularly at a dose that may not impact the general mortality of the individuals, maintaining the population size. This study is intended to ensure the safety of non-target organisms, including humans, exposed to such environmental pollutants unintentionally.

Because of the time-consuming nature of long-term experiments, a number of quick tests using bacteria, plants, rodents, and mammals as test models are in practice. In vivo studies are preferred over in vitro ones as later results are frequently found falsely positive. However, nowadays, in vivo research on large animals like monkeys, rats, guinea pigs, etc., is viewed as unethical. In light of this, insects have been suggested as useful alternative experimental material for such studies. Among insects, mosquitoes are selected, recommended and proved to be a good test model for assessing the clastogenic, mutagenic, and genotoxic potential of different environmental pollutants [25,26,27,28,29,30,31]. A few of the benefits of their selection in these studies include their short life span, easy rearing and maintenance in the laboratory, and the presence of a low diploid number of only six relatively large-sized chromosomes. Recently, these have already been used as test models for determining the genotoxicity of even nanoparticles [32]. The most dangerous metallic compounds have particle sizes in the submicron range of 0.25–5.0 and have been shown to be the most harmful to human health. Nanoparticles, being still smaller in size, are expected to be more hazardous than their larger counterparts due to their enhanced reactivity and penetrability in cells [33]. So, it becomes essential to analyze their genotoxic impact on living organisms and categorize them as safe or dangerous for biodiversity and human health. The main purpose of this study is to assess the genotoxic potential of these nanoparticles by observing their impact on mitotic index and genetic material.

## 2. Materials and Methods

A colony of the mosquito *Culex quinquefasciatus* Say belonging to the family Culicidae and order Diptera was raised and maintained in special mosquito-rearing cages at a constant temperature of 27 ± 2 °C and relative humidity of 75 ± 5% for sufficient and constant availability of the material to carry out various experiments.

### 2.1. Preparation of TiO_2_ Nanoparticles

TiO_2_ nanoparticles used in the present study were synthesized by sol–gel auto-combustion route [34]. Initially, two different solutions, X and Y, were prepared.

1. Solution X: Prepared by mixing titanium (IV) isopropoxide (TTIP, C_12_H_28_O_4_Ti, 97% Sigma Aldrich, Saint Louis, MO, USA) and ethanol (C_2_H_5_OH, Himedia 99.8%) in a ratio of 1:3 for 30 min on a magnetic stirrer (Ambala, Haryana, India).
2. Solution Y: Prepared by mixing double-distilled water (DDW), ethanol and acetic acid glacial AR (CH_3_ COOH) in a ratio of 1:2:7 by stirring for the same time on a magnetic stirrer.
3. Synthesis technique: A milky solution (sol) was obtained by adding, drop by drop, solution X to solution Y. Then, for the formation of gel, this sol was stirred at 25 °C for 10–12 h, maintaining the pH value of approximately 7 by adding ammonium hydro-oxide (NH_4_OH, Nice 30% NH_3_). The gel was dried in an oven maintained at 100 °C for 48–72 h to obtain the crystals. A fine powder was prepared by grinding these crystals using pastel mortar for 45 min. This fine powder was calcined at 450 °C for 2 h in the furnace to obtain titanium dioxide nanoparticles. These nanoparticles’ crystalline form is anatase, having a tetragonal structure, and its average crystallite size is about 10 nm.

### 2.2. Toxicity Assay for Determining LD_20_ of TiO_2_ NPs for Mosquito Culex quinquefasciatus

**Figure 1:**
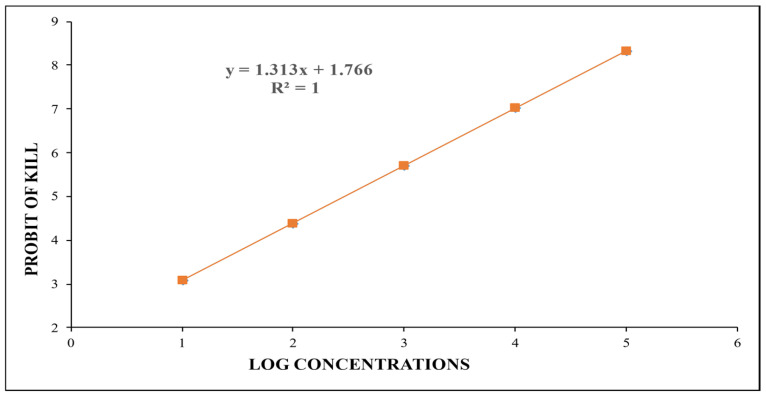
Linear regression line and equation, obtained from mortality data, representing the relationship of Probit of kill and log concentrations of TiO_2_ NPs.

The toxicity assay was conducted by treating 40 early fourth instar larvae of the mosquito to six different doses of these nanoparticles, and a control with an equal number of larvae in an equal volume of distilled water was also maintained. These doses (TiO_2_ NPs suspensions) were prepared by adding 30, 20, 10, 2, 1, and 0.2 mg of the nanoparticles to 20 mL of distilled water in separate sterilized glass vials, and these concentrations were properly vortexed before their application. After 24 h treatment, the larvae were transferred to distilled water and were fed on larval feed made by mixing powdered dog biscuits and yeast in a 3:1 ratio. The mortality data of the treated larvae were recorded (Table 1), and from the percent mortality data, LD_20_ was determined by Probit analysis [35]. From the regression equation y = 1.313x + 1.766 and the regression line so obtained, the value of LD_20_ was calculated, and it came out to be 66.53 µg/mL (Figure 1). 

### 2.3. Selection of Dose for Genotoxic Assays

The selection of a dose is important for quantitative assessment and a more precise evaluation of genotoxic effects because a dose that is too high or too low can give pseudo-positive or negative results, respectively. The dose that does not reduce the population size drastically but does possess some toxicity is usually selected for such assays. Therefore, a toxicity assay was performed to determine the LD_20_ of the nanoparticles for the mosquito *Culex quinquefaciatus*. Out of all the doses used in the toxicity assay, 50 µg/mL dose, the nearest < LD_20_ value, was selected for the various assays to be performed for assessing the genotoxic potential of TiO_2_ NPs. 

### 2.4. Genotoxic Assays

Different assays were performed to assess the genotoxic potential of TiO_2_ nanoparticles and for each assay, 20 early fourth instar mosquito larvae were kept in freshly vortexed 10 mL of the selected dose of TiO_2_ NPs suspension for 24 h. After the treatment, the larvae were shifted to a nutrient medium, which is distilled water with larval feed added to it, so that they could grow and mature into adults. These adults were used to carry out various assays performed to assess the genotoxic potential of these nanoparticles. Three replicates of this treatment were performed, and a control of 20 larvae in 10 mL of distilled water was also maintained.

#### 2.4.1. Mitotic Index Assay

Mitotic slides were prepared from the gonads of 12–15 h old adult mosquitoes from treated as well as control stocks using the method of Crozier [36] with some modifications. After dissecting out gonads in a dissecting medium of 1% sodium citrate solution, these were fixed for 3–5 min in Carnoy’s fixative (ethanol and glacial acetic acid in a 3:1 ratio) and thereafter stained in 2% lacto-aceto-orcein stain for 10–15 min to obtain proper color. A squash preparation of the stained material was made, and the slides were observed under a microscope to study the frequency of dividing cells. 

#### 2.4.2. Chromosomal Aberration Assay

For this assay, chromosomal slides were prepared using the same method but after arresting cell division at the metaphase stage in the gonads by treating the adult mosquitoes with 0.1% colchicine (C_22_H_28_NO_8_, MW 399.44, 98.6% pure procured from Loba Chemie Pvt. Ltd., Mumbai, India) solution about 3–4 h prior to dissection. The slides were screened for chromosomal aberrations, if any, so as to determine genetic damage induced by the nanoparticles. 

#### 2.4.3. Statistical Analysis

Student’s *t*-test was applied to compare the mean percent frequency data of at least three replicates obtained for mitotic indices and chromosomal aberrations induced in the gonadial cells of treated and control mosquitoes. 

## 3. Results

### 3.1. Mitotic Index

**Figure 2:**
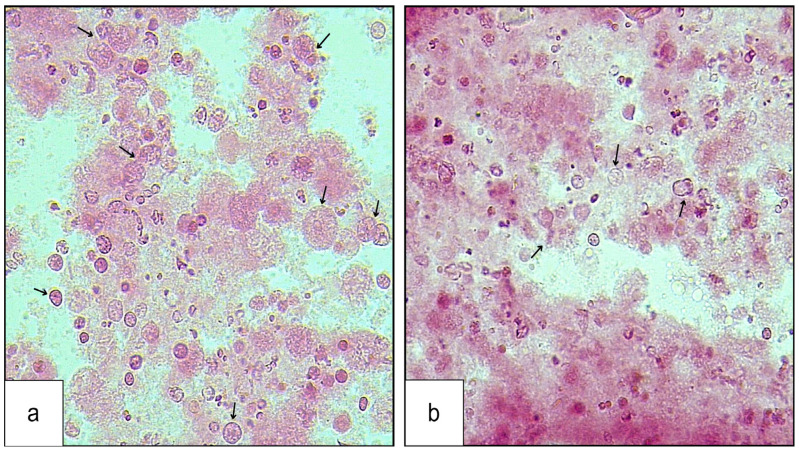
Gonadial cells of adult mosquito *Culex quinquefaciatus*: (**a**) control mosquito with more dividing cells and (**b**) treated mosquito with fewer dividing cells.

About 600–800 cells were studied in each of the three replicates, and the percentage frequency of dividing cells (MI) was calculated. The mean MI values came out to be 52.05 ± 6.22% for treated mosquitoes against 83.63 ± 5.55% in the control (Table 2, Figure 2). The comparison of mean values revealed a statistically significant decline in mitotic index in treated mosquitoes compared to the controls (*p* < 0.05). 

### 3.2. Chromosomal Aberrations

**Figure 3:**
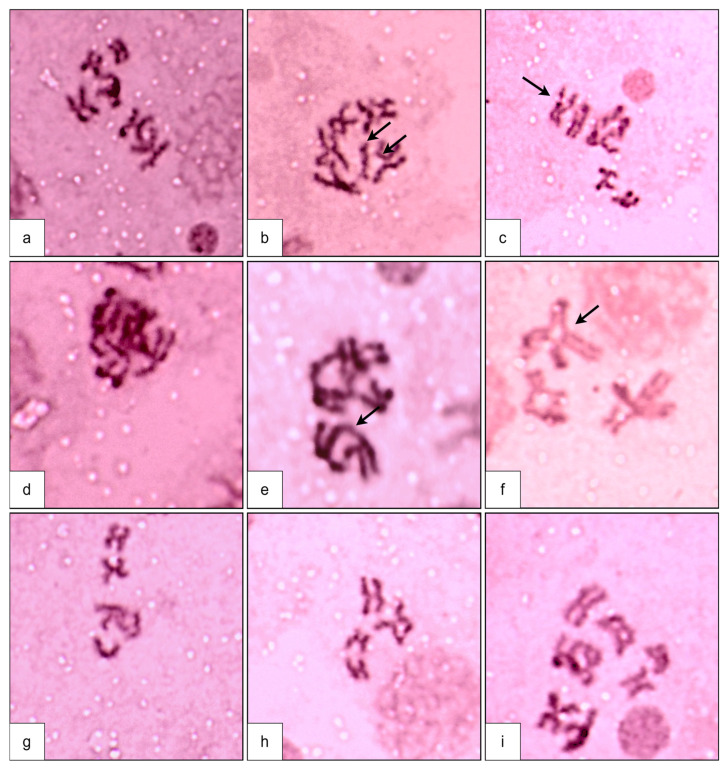
Chromosomal aberrations, structural (**b**–**f**) and numerical (**g**–**i**) ones induced by TiO_2_ NPs: (**a**) normal chromosomal complement (2n = 6), (**b**) chromosomal fragments, (**c**) chromosomal break, (**d**) clumped chromosomes, (**e**) terminal fusion, (**f**) translocation, (**g**) 2n − 1, (**h**) 2n − 2, and (**i**) 2n + 2.

In the present study, both structural as well as numerical changes in the chromosomes after treatment of TiO_2_ nanoparticles were observed. Structural changes include clumping, fragments, terminal fusions, breaks, and translocations of chromosomes (Figure 3), while numerical alterations recorded were aneuploid cells. The hyperploid cells, such as trisomic (2n + 1) and tetrasomic (2n + 2), as well as hypoploid cells with monosomic (2n − 1) and nullisomic (2n − 2) conditions, were observed. An average of 105 cells were studied for chromosomal aberrations in each of the treated and control slots. The percent frequency of total aberrations recorded was 19.32 ± 2.61% in TiO_2_ NPs treated mosquitoes, and this was only 2.52 ± 0.21% in the controls (Table 3). The outcome is statistically significant (*p* < 0.05). Out of the total aberrations induced, the frequency of structural aberrations was found to be 15.20 ± 1.21%, and this value is 1.90 ± 0.06% in controls. The most prevalent structural aberration found was cells with clumped chromosomes, having a frequency of 4.38 ± 0.84%, and the least observed was breaks in chromosomes with a frequency of 1.55 ± 0.47. 

## 4. Discussion

The release of TiO_2_ NPs nanoparticles in the environment, due to their wide range of industrial applications, is increasing and causing a threat to organisms thriving in aquatic as well as terrestrial ecosystems. In order to assess the environmental danger that a particular species is exposed to, it is crucial to understand the effects caused by TiO_2_ NPs as well as the mechanisms underlying their activity, which may be ROS- or non-ROS-mediated [37,38]. The genotoxic potential of TiO_2_ NPs using short-term in vivo assays, viz. mitotic index and chromosomal aberration assays, are studied. Both the assays are performed at a selected dose of <LD_20_. This idea of using <LD_20_ is endorsed by the recommendation of the cell-inhibiting concentration IC_25_ for in vitro cytotoxic assays by Santos-Aguilar and others [39]. Further, El Yamani and associates, in 2017 [40], found these nanoparticles genotoxic at non-cytotoxic concentrations in cultured human lymphoblastoid cells by comet assay. This study also emphasizes the importance of the selection of doses for such studies.

Different researchers reached positive as well as negative outcomes in both in vivo and in vitro experiments performed by different test models after TiO_2_ NPs exposures [21,23,24,37,41,42,43,44]. Contrary results obtained in different studies are obvious because of the use of different concentrations, sizes, and forms of the nanoparticles to assess the genetic damage induced. Donner and others [45] could not find any micronuclei induction in the reticulocytes of rats fed with different doses of these nanoparticles. Lack of absorption from the alimentary tract was explained as a reason for the negative effects observed. However, Gao and coworkers [46] observed an adverse impact on ovarian cells in mice after intragastric infusion of TiO_2_ NPs, and this observation indicates absorption through the digestive tract wall. Migration across cell membranes leading to the uptake and internalization of these particles into cells was reported by others [43,47]. Dorier and associates [48] stressed the role of some carriers and efflux pumps in the uptake of these nanoparticles across the gut epithelium. Most of these nanoparticles are reported to migrate and accumulate in vital organs, induce toxic effects in their tissues, and disturb the metabolic processes of the organism’s body [49,50,51]. In the present study, these particles were found inside the haemocoel of mosquitoes, and the internalization of these particles is further endorsed by positive results obtained in both the parameters carried out to test the genotoxicity of the nanoparticles in question. A statistical increase in chromosomal aberrations over control was observed. Both structural alterations, including chromosomal clumping, fragments, breaks, terminal fusions, and translocations, as well as numerical alterations comprising hyperaneurploids (2n + 1 and 2n + 2) and hypoaneuploids (2n − 1 and 2n − 2) were encountered. The breaks near the terminal ends of chromosomes make them sticky, leading to clumping and terminal fusions. The reason for the occurrence of aneuploids may be due to the abnormal disjunction of chromosomes during their segregation at anaphase stages of mitosis [29]. These results are in conformity with similar observations on germ and bone marrow cells in mice [52,53,54,55]. An increase in chromosomal abnormalities was also shown in cultured human peripheral blood cells after 24 h exposure to these nanoparticles by Patel and others [56]. Further, they also observed double-strand breaks in cultured bone marrow cells of rats, indicating the genetic damage caused by these nanoparticles. The genetic damage induced by NPs may be due to oxidative stress and the generation of cytokines, which harm DNA, lipids, proteins, and carbohydrates [57,58,59,60].

An EFSA [61] panel chaired by Younes in 2021 published a report regarding the safety assessment of titanium dioxide (E171) and concluded that E171 cannot be considered safe as a food additive, even after considering the limited relevance of genotoxic studies with TiO_2_ NPs with size < 30 nm. Although NPs present in E171 food additives are bigger (size > 30 nm) and constitute only a minor proportion of the additive, recently, in 2024, Warheit [62] put forth a view that labeling these particles risky may be an error, as its genotoxicity depends on diverse factors and positive results shown in various such studies are controversial. However, as per the report of Weir et al. [2] in the year 2012, these NPs constitute 36% of this additive, which is used in most common food items like chewing gum, candies, white icing on cakes, etc., repeatedly consumed in large amounts, especially by children, its safety assessment is very crucial as future generations of human beings are at stake. If any agent induces chromosomal aberrations, which are direct evidence of genetic damage, that agent cannot be considered safe. In the present study, too, statistically significant chromosomal aberrations are reported to be induced by these NPs, although having a size < 30 nm. The EFSA panel seems to be right or genuine in labeling E171 as unsafe, especially for its repeated exposures. 

A statistically significant decline in mitotic index in treated mosquitoes compared to the controls (*p* < 0.05) was observed in the present research work. A delay in the G2 to M phase of the cell cycle and a slowdown in the mitotic division were observed [63,64]. A decrease in cell division is also reported by Ferrante and team [65]. The reason for this decline may be the impact of these nanoparticles on the expression of genes involved in the regulation of mitosis [22,66,67]. The cytotoxic effects of nanomaterials have been reported to be due to their interaction with enzymes involved in the regulation of cell division [68]. Sarikhani and others, in the year 2022 [69], suggested a decrease in stem cells due to the induction of apoptosis during the cell cycle as the cause of retardation of cell proliferation leading to a decline in the mitotic index of cells. The induction of cellular apoptosis by TiO_2_ NPs was also shown by other research workers [39,70]. Over the past few decades, the majority of data on the photocatalytic activity of TiO_2_ NPs and their associated phototoxic effects have been acquired at the cellular level [37,71].

Crystalline form and particle size have been shown to influence the genotoxic potential of TiO_2_ nanoparticles. The anatase form of the nanoparticles was found to be more genotoxic than its other forms [72,73]. Gurr and others [74] reported 10–20 nm-sized TiO_2_ nanoparticles-induced genotoxicity in the form of DNA damage and micronuclei formation in human bronchial epithelial cell lines. The reason for this toxicity was found to be oxidative stress, which is evident from their observation of the increased production of hydrogen peroxide in the absence of photo-activation. However, these results were not reported after these cell lines were exposed to 200 to > 200 nm-sized particles under similar conditions. In murine MC3T3-E1 pre-osteoblast cells, the toxicity of the cells treated with 5 nm TiO_2_ NPs exhibited higher levels of lactate dehydrogenase (LDH) release, mitochondrial damage, and apoptosis when compared to those treated with 32 nm TiO_2_ NPs [75]. Some other findings also implied that the smaller the particle size, the more genetic damage is caused [76,77,78]. In the present study, the anatase form of these particles measuring 10 nm was used, and it gave statistically significant positive results, thus confirming the above-mentioned observations. The possible mechanism for genetic damage by titanium dioxide nanoparticles was indicated to be due to the release of reactive oxygen species (ROS), which in turn causes lipid peroxidation [3,44,53,79,80]. Mancuso and team [81] reported that the anatase crystal, the form of the nanoparticles used in the present study, is also able to induce the production of ROS in cells, and this could be the plausible reason for their genotoxic potential.

## 5. Conclusions

From the present study, it is quite evident that TiO_2_ NPs, particularly having a size of about 10 nm, do possess genotoxic potential, as these nanoparticles have been found to induce statistically significant (*p* < 0.05) decline in mitotic index and increase in various structural and numerical chromosomal aberrations over control at a dose less than LD_20_ and are not absolutely safe for use in different consumer products. The present study warrants their careful use and disposal in future. Further, experiments can be conducted to mitigate the harmful impacts of these nanoparticles.

## Figures and Tables

**Table 1:** The percent mortality data of *Culex quinquefaciatus* mosquito after TiO_2_ NPs treatment for 24 h.

Doses		Number of Larvae Treated (n)	Number of Larvae Killed (r)	Percentage Mortality (r/n × 100)
(mg/mL)	(µg/mL)			
1.5	1500	40	36	90%
1.0	1000	40	30	75%
0.5	500	40	22	55%
0.1	100	40	16	40%
0.05	50	40	6	15%
0.01	10	40	3	7.5%
Control		40	2	5%

**Table 2:** Percent frequency of dividing cells in the gonads of TiO_2_ NPs treated and control adult mosquito *Culex quinquefaciatus*.

Treatment	Replicates	Total No. of Cells Observed	No. Of Dividing Cells	% Frequency of Dividing Cells	Percent Mitotic Index (Mean ± S.E.)	‘*t*’-Value
TiO_2_ NPs	R1	788	290	36.80	52.05 ± 6.22	−3.11 *
	R2	661	395	59.75		
	R3	785	468	59.61		
Control	R1	814	720	88.45	83.63 ± 5.55	
	R2	619	571	92.24		
	R3	715	502	70.20		

* The results are statistically significant at *p* < 0.05. The negative value of ‘*t*’ indicates decline in mitotic index from control values.

**Table 3:** Percent frequency of mitotic chromosomal aberrations induced by TiO_2_ NPs in gonadial cells of mosquito *Culex quinquefaciatus*.

Treatment	Chromosomal Aberrations												
	Structural Aberrations (SA)					Numerical Aberrations (NA)				Total Structural Aberrations	Total Numerical Aberrations	Total Aberrations	‘*t*’-Value
						Aneuploids							
	Clumped Chromosomes	Fragments	Terminal Fusions	Breaks	Translocations	(2n + 1)	(2n + 2)	(2n − 1)	(2n − 2)	(SA)	(NA)	(SA + NA)	
TiO_2_ NPs	4.38 ± 0.84	2.55 ± 0.55	3.52 ± 0.99	1.55 ± 0.47	3.17 ± 0.27	0.95 ± 0.46	1.90 ± 0.46	0.62 ± 0.25	0.62 ± 0.25	15.20 ± 1.21	4.12 ± 1.40	19.32 ± 2.61	2.63 *
Control	0.61 ± 0.25	0.32 ± 0.26	0.67 ± 0.54	0.00	0.28 ± 0.23	0.00	0.00	0.61 ± 0.25	0.00	1.90 ± 0.06	0.61 ± 0.25	2.52 ± 0.21	

* The data are mean ± S.E. of three replicates and are statistically significant at *p* < 0.05.

## Data Availability

The data presented in this study are available on request from the corresponding authors.
